# Difficulties in Recognising Dynamic but not Static Emotional Body Movements in Autism Spectrum Disorder

**DOI:** 10.1007/s10803-021-05015-7

**Published:** 2021-04-17

**Authors:** Noemi Mazzoni, Paola Ricciardelli, Rossana Actis-Grosso, Paola Venuti

**Affiliations:** 1 OFDLab - Department of Psychology and Cognitive Science, University of Trento, Via Matteo del Ben, 5B, 38068 Rovereto, Italy; 2 Department of Psychology, University of Milano - Bicocca, Milan, Italy; 3 Milan Centre for Neuroscience, University of Milano-Bicocca, Piazza dell’Ateneo Nuovo 1, 20126 Milan, Italy

**Keywords:** Autism spectrum disorder, Emotion recognition, Biological motion, Static and dynamic body stimuli, Emotional body movement

## Abstract

In this study, we investigated whether the difficulties in body motion (BM) perception may led to deficit in emotion recognition in Autism spectrum disorder (ASD). To this aim, individuals with high-functioning ASD were asked to recognise fearful, happy, and neutral BM depicted as static images or dynamic point-light and full-light displays. Results showed slower response times in participants with ASD only in recognising dynamic stimuli, but no group differences in accuracy. This suggests that i) a deficit in action chaining mechanism in ASD may prevent the recognition of dynamic BM automatically and rapidly, ii) individuals with ASD and high cognitive resources can develop alternative—but equally successful—strategies to recognise emotional body expressions. Implications for treatment are discussed

**Supplementary Information:**

The online version contains supplementary material available at 10.1007/s10803-021-05015-7.

Autism Spectrum Disorder (ASD) is a neurodevelopmental disorder characterised by persistent deficits in social communication and social interaction, in social-emotional reciprocity, and in non-verbal communicative behaviour (DSM 5, APA 2013). The ability to recognise emotions is a core deficit of this condition and has been mentioned as distinctive since its original description (Kanner 1943). However, despite decades of research, the exact nature of the emotional deficits in ASD remains unclear. Emotion recognition in ASD has traditionally been investigated using facial expression (see Harms et al. 2010 for a review). A meta-analysis concluded that emotion recognition through facial expressions is generally impaired in ASD (Uljarevic & Hamilton, 2013) providing evidence against a deficit in decoding one particular emotion.

Although the face is considered seminal in conveying emotions, other nonverbal channels, such as voice and body movement (BM), contribute significantly to communicate others’ feelings and mental states. In particular, it has been demonstrated that BM is as important as face (Gelder, 2006; Gelder et al. 2010) – or even more (Aviezer & Todorov, 2015; Stock et al. 2007)—in conveying emotional cues.

## Recognition of Emotional BM in ASD from Dynamic Stimuli

In individuals with ASD, the difficulty in processing social cues is not limited to facial stimuli, but encompasses also information from BM (Kaiser & Pelphrey, 2012). Thus, it has been suggested that the difficulties in social interaction in ASD could be in part explained by deficit in BM processing (Kaiser et al. 2010; Pavlova, 2012). Traditionally, the decoding of BM has been investigated using the so called point-light displays (PLDs) that consist in few markers placed over the major joints of a moving actor filmed in a dark set (Johansson, 1973). As a result, in PLDs the kinematic information is isolated from the other visual cues (e.g., colour, shape, and texture). This offers a unique opportunity to study the role of BM in conveying social meaningful cues. When *non-emotional* PLDs were presented (e.g. walking), conflicting results have been reported: some studies found impairment associated with ASD (Annaz et al. 2010; Blake et al. 2003; Kaiser & Pelphrey, 2012; Mazzoni et al. 2020), while others did not (Freitag et al. 2008; Herrington et al. 2007; Murphy et al. 2009). Conversely, deficits in recognizing *emotional* PLDs have been consistently reported throughout development, in children (Mazzoni et al. 2020; Moore et al. 1997), adolescents (Hubert et al. 2007; Parron et al. 2008), and adults (Atkinson, 2009; Nackaerts et al. 2012) with ASD (but see Actis-Grosso et al. 2015).

Note that, although PLDs stimuli offer the advantage to isolate the motion information from the other visual cues, they are not representative of natural social interaction. Indeed, in daily life the representation of BM is never limited to its pure kinematic features – as it is in PLDs—but it is always associated with the vision of the body form. This should be considered in research aimed to explain social interaction difficulties in ASD. To overcome this issue, Atkinson and collaborators (Atkinson, 2009) compared the recognition of emotional BM between traditional PLDs and more naturalistic full-light displays (FLDs) of the same BM. They found that adults with ASD were less accurate than TD controls in identifying happiness, anger, and disgust, and marginally less accurate in identifying fear and sadness, both when presented as FLDs and PLDs.

Another study (Mazzoni et al., 2020) has recently replicated this result in children with ASD with and without intellectual disability (ID). Using the same stimuli as Atkinson et al. (2009), the authors found a difficulty in recognizing the emotional content of BM in children with ASD irrespective of their IQ, either when presented as PLDs or FLDs. These results suggest that a deficit in emotion recognition might be related to abnormalities in BM processing, irrespective of its visual representation. Moreover, in this study the impairment in children with ASD was not limited to emotional movements, but encompasses also neutral stimuli. This is in line with the hypothesis that poor motion perception may interfere with the recognition of emotional whole-BM (Dakin & Frith, 2005) and suggests that the difficulties in social interaction in individuals with ASD might be related to a general impairment in processing the BM, rather than being related to the processing of a specific emotion or class of stimuli—consistently with results in emotional faces (Uljarevic & Hamilton, 2013).

### Recognition of Emotional BM in ASD from Static Stimuli

If the difficulty in recognizing the emotional content of BM in ASD is related to the processing of BM information, it is reasonable to suppose that the recognition of static body postures will be preserved. Whereas, if the impairment encompasses also the recognizing the emotional static body postures, this will suggest a deficit related to the decoding of emotional content.

The results about the recognition of emotional BM using static stimuli are mixed. Some studies found no differences between children (Peterson et al. 2015) and adults (Libero et al. 2014) with and without ASD. In this latter study, although no differences were observed at the behavioural level, the authors found significant differences in functional connectivity between groups in motor-related areas (i.e., ventral premotor cortex, inferior frontal gyrus, and superior parietal areas) that are part of the putative mirror neuron system.

In contrast, other studies showed poorer identification of fearful body postures in participants with ASD, compared to TD controls. For instance, in adolescents with Asperger’s Syndrome (AS), Doody and Bull (2013) found poorer accuracy in verbal labelling of fearful stimuli (but no different accuracy in a nonverbal matching of the same stimuli) and longer response times for angry body posture in a nonverbal matching task, suggesting an impairment specific to threating stimuli. Consistently, Philips and collaborators (Philip et al., 2010a) compared the recognition of emotional images of faces, videos of bodies, and voices and found that participants with ASD were less accurate than TD in all the stimulus categories. Finally, Hadjikhani and collaborators (2009) found that adults with ASD were more accurate than TD in recognizing neutral body postures, but less accurate in recognizing the fearful ones. Furthermore, unlike TD participants, in participants with ASD the vision of fearful postures did not elicit response in visuo-motor areas (intra-parietal lobule and inferior frontal gyrus) and failed to elicit differential brain activity for emotional and neutral body postures.

### Possible Reasons of Inconsistency in Research on BM in ASD

The inconsistent results in research on static BM in ASD may be explained by some methodological differences. For instance, very small samples size (e.g. Hadjikhani et al., 2009), unbalanced numerosity between groups (Peterson, Slaughter, & Brownell, 2015), and inconsistency in matching criteria may led to failure in replicating results in heterogeneous clinical conditions, such as ASD. Specifically, in two studies the group of participants were not IQ matched (Hadjikhani et al., 2009; Philip et al., 2010b). The most of other studies used verbal (VIQ) or full-scale IQ (FSIQ) as group-matching criteria (Doody & Bull, 2013; Libero et al. 2014; Peterson et al. 2015). Critically, in FSIQ the verbal and nonverbal performance are averaged together and this may mask group-differences in cognitive profile that, in turn, can inflate the results. Moreover, deficits in facial (Bormann-Kischkel et al. 1995; Macdonald et al., 1989; Tantam et al. 1989) and bodily (Mazzoni et al. 2020) emotion recognition in ASD seems to emerge clearly when the nonverbal IQ was used as matching criteria, but to a lesser extent when verbal IQ was used (Castelli 2005; Davies et al. 1994; Loveland et al. 1997). For these reasons, in the present study, participants were matched according to nonverbal IQ and performed a nonverbal recognition task.

Inconsistency in results may also rely to the stimuli’s features, such as differences in emotional content and in lower-level visual features. Indeed, some studies presented emotional but not neutral BM (Doody & Bull, 2013; Peterson et al. 2015; Philip et al., 2010b), or neutral and negative but not positive expressions (Doody & Bull, 2013; Hadjikhani et al., 2009). Besides, portray of static emotional BM have been vary, including black and white silhouettes (Libero et al., 2014), avatars (Doody & Bull, 2013), and naturalistic images (Hadjikhani et al., 2009; Peterson, Slaughter, Brownell, et al., 2015). Possibly, the great number of details in naturalistic stimuli can have diverted attention from emotional content in ASD participants. To limit this bias, in the present study changes of not relevant features were minimized (e.g., showing actors wearing similar dresses).

Finally, inconsistency in results may be due to the type of task (e.g., verbal vs nonverbal, free vs forced choice labelling). Forced-choice paradigm (Libero et al., 2014; Peterson et al. 2015; Philip et al., 2010b) required to select a response option even though the expressed emotion is not recognized. This may artificially inflate the agreement in ASD participants and lead to different neurological activity in spite of comparable behavioural performance (Libero et al. 2014). Furthermore, in nonverbal match-to-sample task (Doody & Bull, 2013; Hadjikhani et al. 2009), participants can match the response options according to the visual features (e.g., limbs/trunk position) irrespective of emotion recognition. To prevent participants from relying on visual features, in this study we asked participants to match emotional BM with the respective facial expression.

### Comparison Between Static and Dynamic Body Stimuli in ASD

Crucially, none of the abovementioned studies compared static and dynamic bodily expressions. To the best of our knowledge, only one study has directly compared the recognition of static and dynamic body stimuli in participants with ASD (Weisberg et al., 2014). In a fMRI task, the authors presented static images, full-colour videos, and PLDs depicting neutral human actions (social) or tools (non-social). Behavioural results revealed no group differences in distinguishing tools from human stimuli, regardless of whether the stimuli were static or dynamic. At the neural level, in TD but not in ASD group, the authors found a heightened response to social compared to non-social stimuli, specifically for dynamic ones. Notably, the human movements used in this study were emotionally neutral. To date, the direct comparison of static and dynamic bodily expressions in ASD remains unexplored.

To fill this gap, in the present study we investigated the role of motion cues (implied vs dynamic) in modelling the recognition of emotional content of BM in ASD. To this aim, we asked a group of adolescents and adults with high-functioning ASD (HFA—namely, without intellectual disability) to recognise static images and dynamic FLD and PLD videos depicting happy, fearful, and neutral body movements. Building on previous studies’ criticalities, we used a nonverbal recognition task and, consistently, we matched ASD and TD groups according to nonverbal IQ. We hypothesise that, if the difficulty in recognizing the emotional content of BM in ASD is related to the processing of motion information, we will find no group differences in recognizing static body postures, but impaired recognition of dynamic body stimuli in participants with ASD.

## Methods

### Participants

20 participants with HFA (age range 13–27 years, age mean = 19.85; age sd = 7.77) and 20 typically developed controls (age range 14–26 years, age mean = 20.44, age sd = 5.27) took part in this study (Table 1). This numerosity was determined taking into account the sample size of previous studies in the field (Atkinson, 2009; Annaz et al., 2012; Doody & Bull, 2013; Hubert et al., 2007; Parron et al. 2008; Philip et al. 2010). Participants with and without ASD were matched according to gender, age, and nonverbal IQ measured with the Raven’s progressive matrices (Raven, 1936) (Table 1).

**Table 1:** The participants female:male ratio, together with mean, and standard deviations (sd) of Age and IQ in ASD and TD participants of the main experiment are reported

Group	ASD	TD	t-test
	Mean (sd)	Mean (sd)	p-value
F:M ratio	0:20	2:20	–
Age	19.85 (7.77)	20.44 (5.27)	0.782
IQ	116.85 (10.03)	123.45 (8.68)	0.063

The t-test column shows the results of between group comparisons

All participants had normal or correct-to-normal vision. Participants with ASD were recruited at the Observation, Diagnosis, and Education Laboratory (University of Trento) and met the criteria specified in DSM-IV (American Psychiatric Association 2006) or DSM-5 (American Psychiatric Association 2013), or the Autism Diagnostic Observation Schedule (ADOS—Lord et al. 2000), or the Autism Diagnostic Interview (ADI—Lord, Rutter, & Le Couteur, 1994). Data on socioeconomic status were not recorded. Before the experiment, all participants received an exhaustive explanation of the experimental procedure and written informed consent was provided by them or their parents, according to the Declaration of Helsinki. The study was approved by the ethical committee of the University of Milano-Bicocca.

### Stimuli

We presented neutral and emotional whole-body movements depicted as static body images (SB), and dynamic video-clips of full-light (FLDs) and point-light (PLDs) displays. The final set of stimuli included 24 stimuli (8 neutral, 8 fearful, 8 happy) for each category.

### SB

**Fig. 1:**
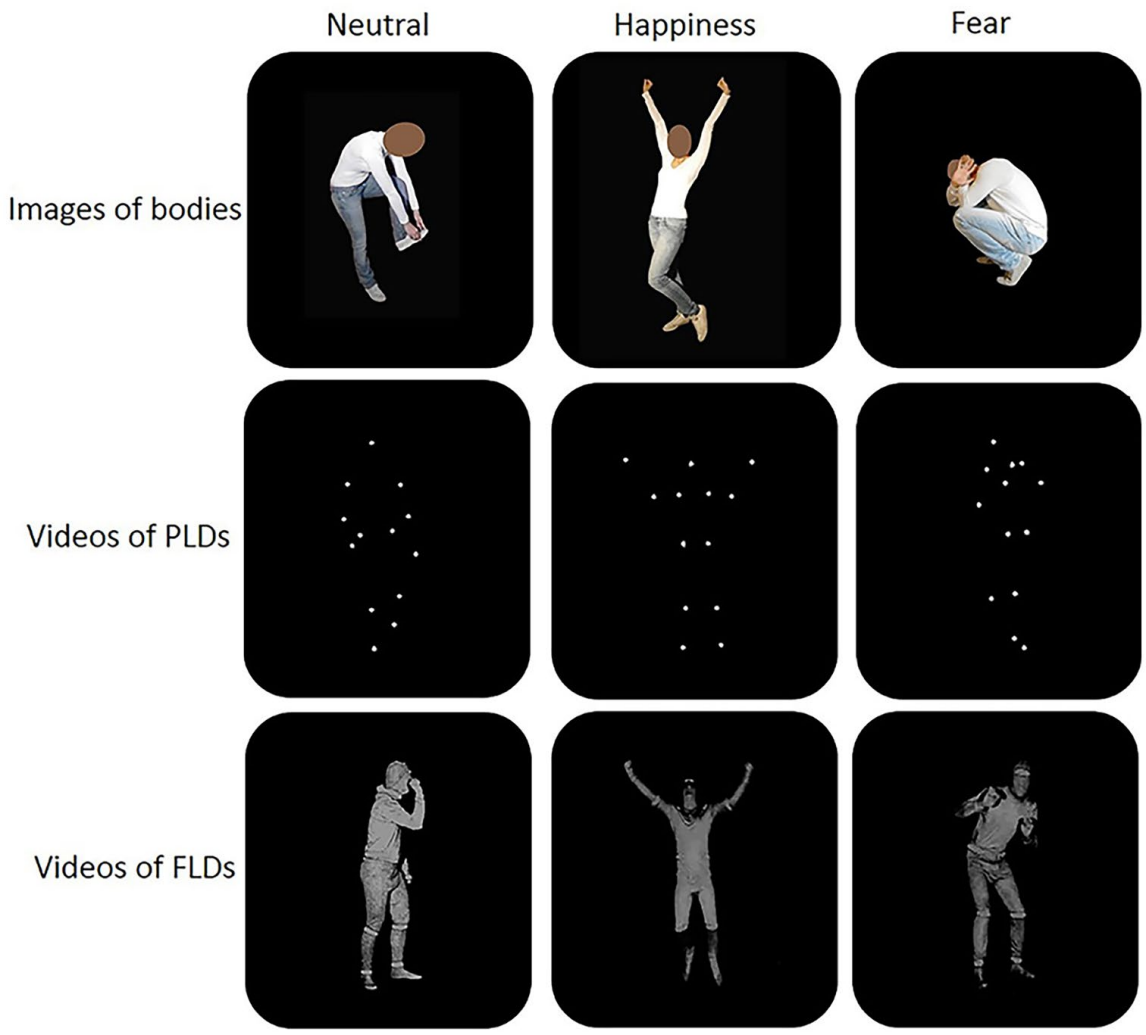
Example of the stimuli presented in the main experiment

The static images were created by our group of researchers (Supplementary materials). All the actors (4 females and 4 males) wore a white T-shirt with long sleeves, blue jeans, and sneakers. All the pictures were edited with Photoshop: brightness, number of pixels and dimension were equalised among all the pictures, the background colour was turned to black, and the face was blurred (Fig. 1). The recognizability of the stimuli was assessed in a Validation study that involved 20 TD adults (16 males and 4 females, age mean(sd) = 24.65(1.66), Supplementary materials).

For the main study, we selected a total of 24 images (8 happy, 8 fearful, 8 neutral) whose accordance about the expressed emotion ranged between 80 and 100% (that is, at least 80% of participants responded that that images depicted a specific emotional expression—e.g., fear—or a specific action—e.g., push). For the emotional expressions, only the fearful and happy stimuli whose intensity was rated above 4.7 were selected; for the neutral actions, only the stimuli that were never confounded as an emotion were selected. The actors’ gender was equally represented (4 males, 4 females). The final dataset of SB images is available on request from the corresponding author.

### PLDs and FLDs

The dynamic stimuli consisted in short video clips depicting happy, fearful, and neutral whole-BM represented as FLDs and PLDs. The stimuli were selected from a wider dataset realised by Atkinson and collaborators (Atkinson et al. 2004, 2012). The FLDs consisted of 3 s digital movies depicting a grey-scale actor moving against a black background. The actors’ face was covered. The PLDs lasted 2 s and consisted of 13 lighting dots placed over the main joints of the actor, moving against a black background, and were created by converting the FLDs stimuli to PLDs (Atkinson et al., 2012). The stimuli were created this way by Atkinson and collaborators and we maintained the original timing of presentation. The actors’ gender was equally represented (4 males, 4 females). Examples of the stimuli can be viewed in Fig. 1 and at https://atkinsonap.github.io/stimuli/.

### Main Experiment Procedure

Participants were tested individually in a quiet room. They sat in front of a computer monitor, at a distance of 60 cm. The experimenter sat next to the participant for the entire duration of the study. Participants were presented with fearful, happy, or neutral whole-body movements depicted as SB, FLDs, and PLDs. In total, each participant saw 24 PLDs, 24 FLDs, and 24 images of whole-body expressions (8 videos or images in every emotional category). The three stimulus categories were presented in three separated blocks, each block lasted around eight minutes. Participants could take a break between the blocks, if needed. The order of blocks was counterbalanced between participants. Within each block the stimuli were presented randomly. Every block started with few practice trials. Every trial started with a one-second fixation cross, then the stimulus was presented for 2 s (PLDs) or 3 s (FLDs and static images). The question “Which emotion was expressed?” was presented on the top of the screen and lasted until participants gave the response. Sticky emoticons of happy, fearful, and neutral facial expressions were placed on the response keys. Participants were asked to categorise the observed stimulus by pressing the key with the corresponding emoticon, as accurately and fast as possible. Accuracy and response times (RT) were recorded. Responses were collected by keyboard; the order of key-emotion correspondence was randomised across participants. Stimulus presentation was controlled and behavioural responses were recorded with E-Prime 2.0 software (Psychology Software Tools, Inc). At the end of the experiment, participants were administered with the Matrix of Raven test to assess the nonverbal IQ.

### Statistical Analysis

All the analyses were performed using R, version 3.6.1 (R Core Team, 2019).

#### SATO

**Fig. 2:**
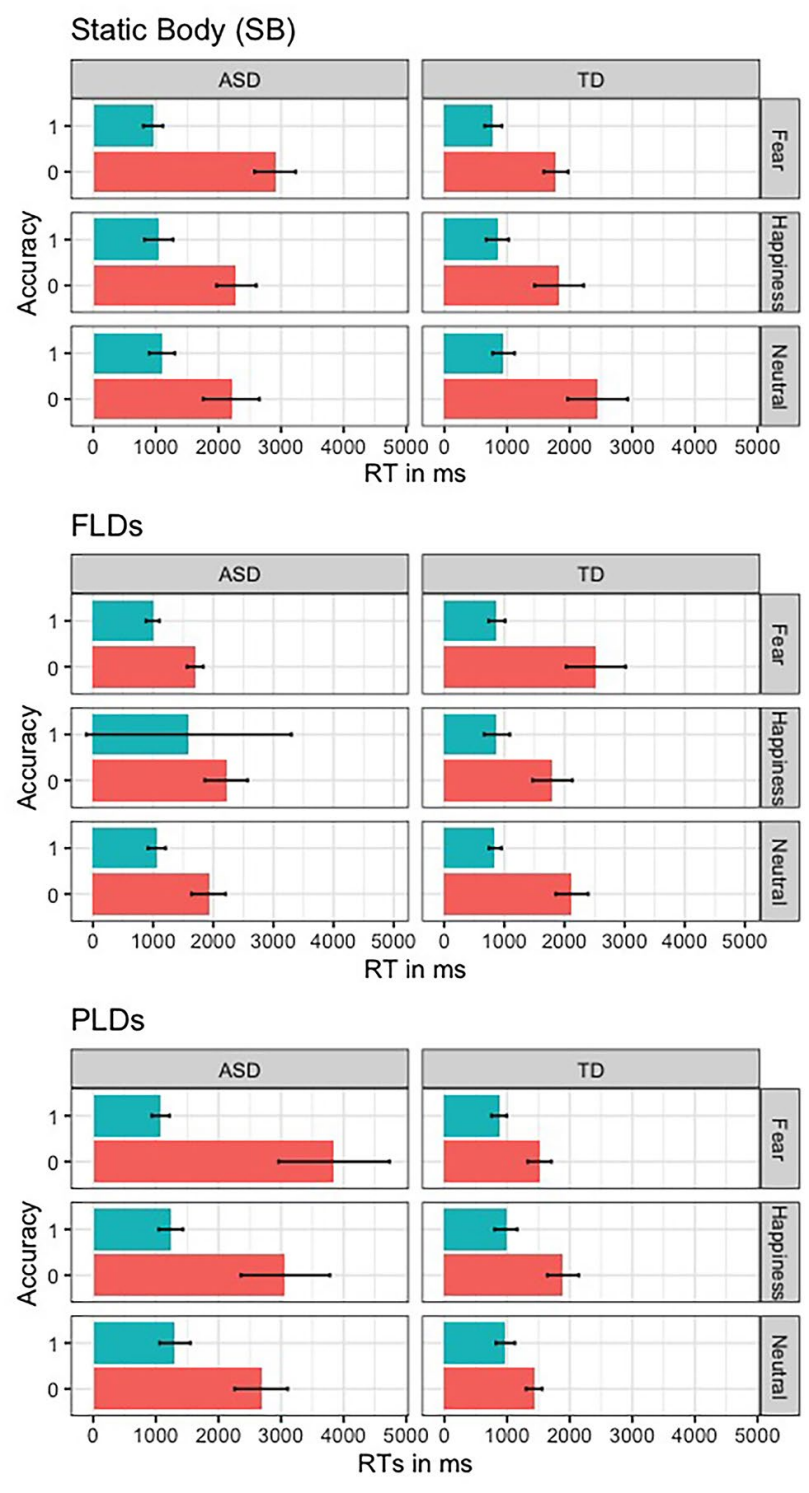
Speed-accuracy trade-off (SATO): The figure represents the mean RTs of the correct (blue) and incorrect (red) responses, relative to the three emotions (fear, happiness, and neutral, in row) in the two groups (ASD and TD, in columns)

Prior to the analysis, we investigated the speed-accuracy trade-off (SATO). To this aim, we plotted the RTs in ms against the percentage of Accuracy for the two groups in recognizing the emotions with different Displays (Fig. 2). Visual inspection of the plot shows that the incorrect answers corresponded to greater RTs in both groups, suggesting that the greater number of errors in participants with ASD was not due to a tendency in responding faster compared to TD ones.

#### Analysis of Accuracy

Percentages of Accuracy are summarised in Supplementary materials. The Accuracy’s distribution violated the assumption of normality (assessed with Shapiro–Wilk normality test) in all the type of stimuli (*BS*: W = 0.695, p-value < 0.00; *FLDs*: W = 0.790, p-value < 0.001; *PLDs:* W = 0.839, p-value < 0.001). We perform a Generalised linear mixed model analysis (function glmer, lme4 package; Bates, Mächler, Bolker, & Walker, 2015) of the relationship between accuracy and emotions, separately in the three classes of stimuli. The models were fitted by maximum likelihood (Laplace Approximation), using binomial distribution. We used *mixed* generalised linear model because our experiment included repeated measures (all participants were presented with the three types of stimuli and the three emotional contents). As fixed effects, we specified Emotion (fear, happiness, neutral) and Group (ASD and TD), with interaction term. As random effects, we included intercepts for subjects as well as by-subject random slopes for the effect of Emotion [example of the syntax *ACC* ~ emotion*group + *(0* + *emotion|subject)]*. Results of the models were then entered in an Analysis of Deviance Table (function Anova, *car* package) using Type III Wald chi-square tests (Fox and Weisberg, 2019).

#### Analysis of Response Times

In the analysis of the Response Time (RTs) only the correct responses were considered. For each group, in every display type separately, the outliers were calculated according to the Tukey’s method (Hoaglin, 2003; Ratcliff, 1993; Rousseeuw & Leroy, 1987) and were discharged, for a total of 4.37% of the SB images, 4.4% of FLDs, and 6.5% of PLDs discharged in ASD group; 4.79% of BS images, 5.58% of FLDs, and 7.29% of PLDs discharged in TD group. To normalise the distribution, the natural logarithms transformation of the averaged RTs was performed and the transformed data were used as dependent variable (logRT). We perform linear mixed model analyses (function lmer, lme4 package) of the relationship between logRT and emotions, separately in the three class of stimuli. The models were fitted by REML, t-tests used Satterthwaite’s method. As fixed effects, we specified Emotion (fear, happiness, neutral) and Group (ASD, TD), with interaction term. As random effects, we included intercepts for subjects and by-subject random slopes for the effect of Emotion [example of the syntax *logRT* ~ emotion*group + *(0* + *emotion|subject)]*. Results of the models were then entered in an Analysis of Deviance Table (function Anova, *car* package) using Type III Wald chi-square tests. Visual inspection of residual plots did not reveal any obvious deviations from homoscedasticity or normality.

## Results

### Accuracy

#### SB

Analysis of variance of type III test of fixed effects (Supplementary materials) showed a significant main effect of Emotion (χ^2^ = 8.405, df = 2, p < 0.015), and no significant effect of Group or interaction Emotion*Group. The generalised linear mixed (glm) model (AIC = 652.5, deviance = 628.5, df-resid = 1908) showed a significantly greater effect for fearful SB compared to neutral (Estimate = -3.39581, StError = 1.26451, Z = -2.685, p = 0.007) and -marginally- to happy stimuli (Estimate = -2.46867, StError = 1.31412, Z = -1.879, p = 0.060) (Table 2). A relevel of the model (with happiness set as reference level) showed no significant differences between the effect of happiness and neutral BS either.

**Table 2:** Results of the generalised mixed models computed on the accuracy in static body (SB), full-light (FLDs) and point-light (PLDs) stimuli (SE indicates the standard errors, CI the confidence interval, Sig the significant results)

Term	Estimate	SE	Z value	p-value	CI_2.5	CI_97.5	Sig
Static Body (SB)							
(Intercept)	6.250	1.320	4.735	0.000	2.529	4.256	**
Emotionh	− 2.469	1.314	− 1.879	0.060	− 1.683	0.192	
Emotionn	− 3.396	1.265	− 2.685	0.007	− 1.282	0.890	*
GroupTD	− 0.241	1.277	− 0.188	0.851	− 1.026	1.252	–
Emotionh:groupTD	0.072	1.210	0.060	0.952	− 1.792	0.634	–
Emotionn:groupTD	0.559	1.205	0.464	0.642	− 1.156	1.709	–
FLDs							
(Intercept)	3.867	0.418	9.253	0.000	2.529	4.256	**
Emotionh	− 0.860	0.531	− 1.618	0.106	− 1.683	0.192	–
Emotionn	− 0.086	0.578	− 0.150	0.881	− 1.282	0.890	–
GroupTD	− 0.575	0.497	− 1.156	0.248	− 1.026	1.252	–
Emotionh:groupTD	0.885	0.663	1.333	0.182	− 1.792	0.634	–
Emotionn:groupTD	0.160	0.667	0.240	0.810	− 1.156	1.709	–
PLDs							
(Intercept)	3.392	0.441	7.700	0.000	2.529	4.256	**
Emotionh	− 0.745	0.478	− 1.558	0.119	− 1.683	0.192	–
Emotionn	− 0.196	0.554	− 0.354	0.723	− 1.282	0.890	–
GroupTD	0.113	0.581	0.194	0.846	− 1.026	1.252	–
Emotionh:groupTD	− 0.579	0.619	− 0.936	0.349	− 1.792	0.634	–
Emotionn:groupTD	0.277	0.731	0.379	0.705	− 1.156	1.709	–

Asterisks significance: ^*^ indicates p-value comprises between 0.05 and 0.001; ^**^ indicates p-value < 0.001

#### FLDs

**Fig. 3:**
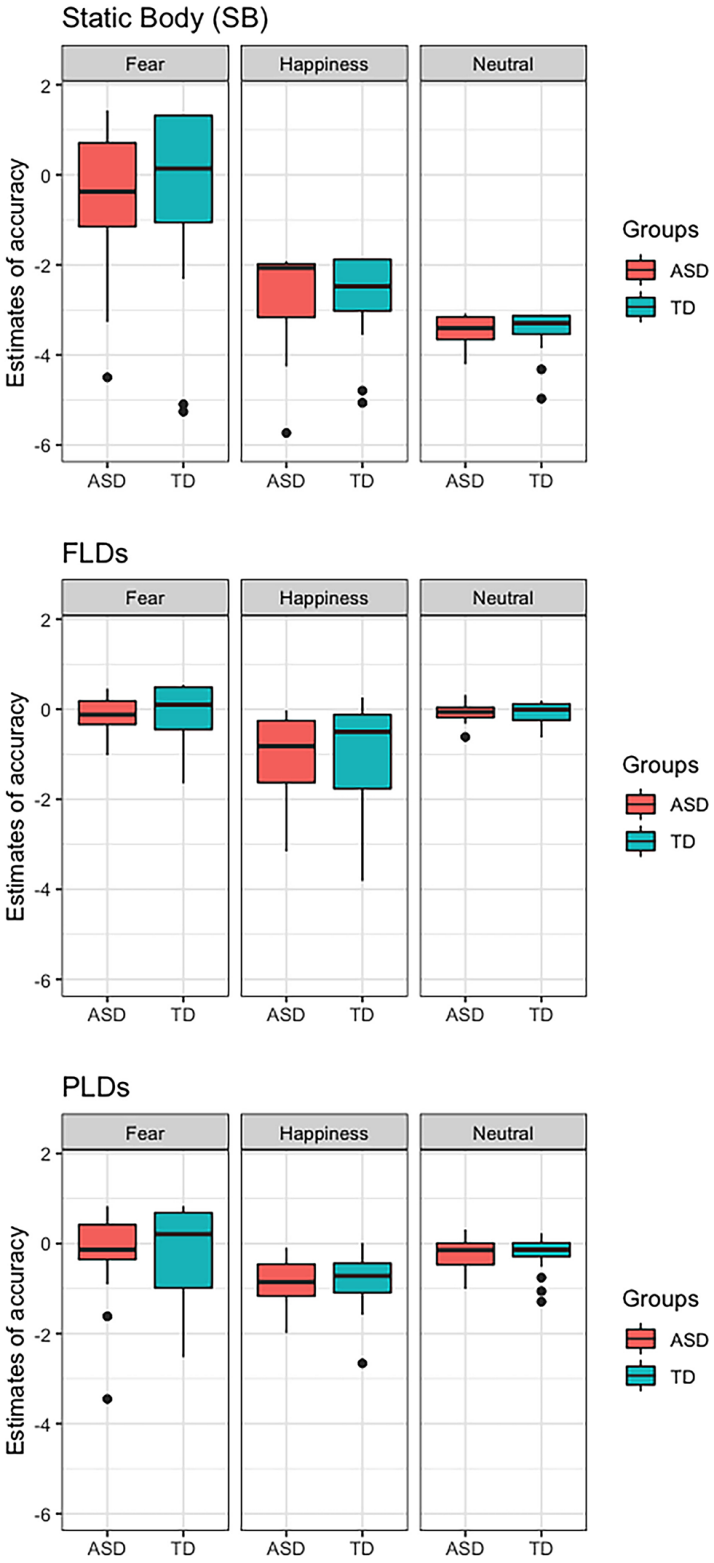
The figure shows the estimated values of accuracy calculated by the generalised lineal mixed models in static body images (SB—top plot), full-light displays (FLDs – central plot), and point-light displays (PLDs – bottom plot). The groups of individuals with ASD and TD are represented in red and blue, respectively

Statistics of type III test of fixed effects of the glm model (Supplementary materials) showed no significant effects, nor any significant effect emerged from the glm model (AIC = 903.6, deviance = 879.6, df-resid = 2388) (Table 2) (Fig. 3).

#### PLDs

Statistics of type III test of fixed effects of the glm model (Supplementary materials) showed no significant effects, nor any significant effect emerged from the glm model (AIC = 1122.5, deviance = 1098.5, df-resid = 2368) (Table 2).

### Response Times (RTs)

#### SB

**Fig. 4:**
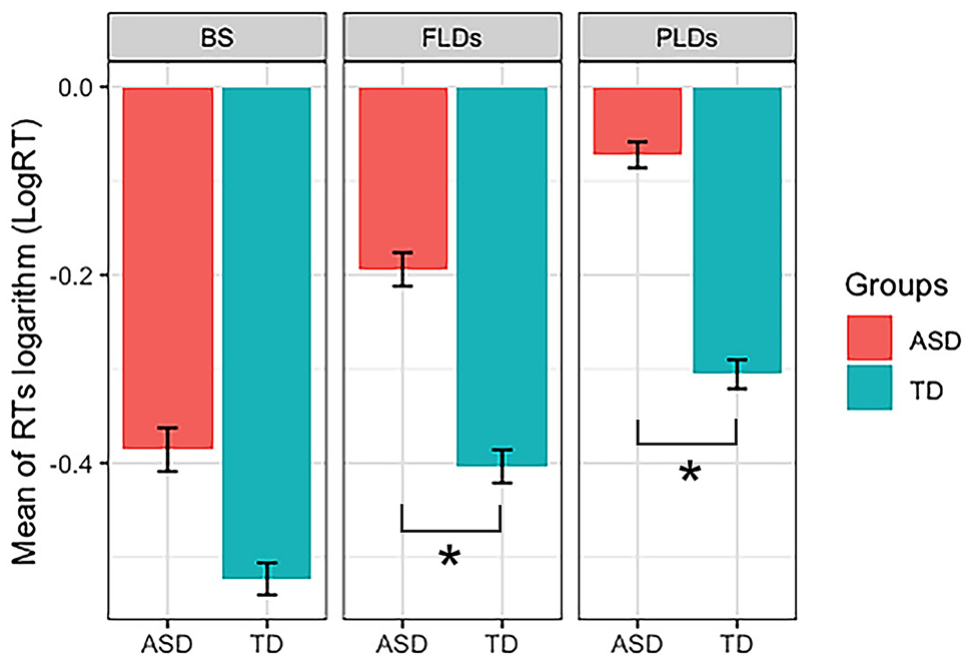
﻿The figure shows the differences between the two groups (ASD and TD groups are represented in red and blue, respectively), in the three display types (SB: static body images; FLDs: full-light displays; PLDs: point-light displays). On the y axis are represented the Logarithm transformation of RTs (LogRT). Stars indicates significant results

All the effect of the model resulted not significant (Table 3). Statistics with type III test of fixed effects are summarised in Supplementary materials. The addition of IQ as covariate did not change the results (Fig. 4).

**Table 3:** Results of the linear mixed models computed on the logRT in static body (SB), full-light (FLDs) and point-light (PLDs) stimuli

Term	Estimate	SE	df	T value	p-value	CI_2.5	CI_97.5	Sig
Static Body (SB)								
(Intercept)	− 0.493	0.094	37.029	− 5.258	0.000	− 0.311	− 0.055	**
Emotionh	0.017	0.045	37.239	0.383	0.704	− 0.015	0.172	–
Emotionn	0.038	0.052	34.790	0.733	0.468	− 0.045	0.110	–
GroupTD	− 0.129	0.133	36.994	− 0.975	0.336	− 0.459	− 0.097	–
Emotionh:groupTD	0.012	0.064	37.489	0.182	0.857	− 0.128	0.138	–
Emotionn:groupTD	− 0.033	0.073	34.757	− 0.445	0.659	− 0.024	0.195	–
FLDs								
(Intercept)	− 0.297	0.078	38.165	− 3.810	0.000	− 0.311	− 0.055	**
Emotionh	0.001	0.055	36.726	0.019	0.985	− 0.015	0.172	–
Emotionn	0.061	0.045	38.147	1.367	0.180	− 0.045	0.110	–
GroupTD	− 0.216	0.110	38.318	− 1.958	0.058	− 0.459	− 0.097	
Emotionh:groupTD	0.001	0.077	36.942	0.013	0.990	− 0.128	0.138	–
Emotionn:groupTD	0.002	0.063	38.465	0.025	0.980	− 0.024	0.195	–
PLDs								
(Intercept)	− 0.183	0.065	37.856	− 2.808	0.008	− 0.311	− 0.055	*
Emotionh	0.078	0.048	39.261	1.639	0.109	− 0.015	0.172	–
Emotionn	0.032	0.040	36.954	0.821	0.417	− 0.045	0.110	–
GroupTD	− 0.278	0.092	37.939	− 3.010	0.005	− 0.459	− 0.097	*
Emotionh:groupTD	0.005	0.068	39.903	0.073	0.942	− 0.128	0.138	–
Emotionn:groupTD	0.086	0.056	36.832	1.536	0.133	− 0.024	0.195	–

Asterisks significance: ^*^ indicates p-value comprises between 0.05 and 0.001; ^**^ indicates p-value < 0.001

SE indicates the standard errors, CI the confidence interval, Sig the significant results

#### FLDs

Anova with type III test of fixed effects (Supplementary materials) showed a significant effect of Group (χ^2^ = 3.83, df = 1, p = 0.05), with slightly faster RT in participants with TD compared to ASD (β = -0.216, SE = 0.110, t-value = -1.958, p-value = 0.0575). All the other effects resulted not significant (Table 3). The significance of the effect of Group increased if the IQ was added as covariate (χ^2^ = 4.19, df = 1, p = 0.04), while the other effects remained not significant. The two models were equivalent (Supplementary materials).

#### PLDs

The analysis of variance with type III test of fixed effects (Supplementary materials) revealed a significant effect of Group (χ^2^ = 9.063, df = 1, p = 0.003), with faster RT in participants with TD compared to ASD (β = -0.278, SE = 0.092, t-value = -3.010, p-value = 0.005). All the other effects resulted not significant (Table 3). The addition of IQ as covariate did not change the results.

## Discussion

The aim of the present study was to investigate the ability of individuals with high-functioning ASD (HFA) to recognise fearful, happy, and neutral body movement (BM) when represented as static or dynamic stimuli. Taken together, our results showed no differences in accuracy between participants with and without ASD, either when the BM were displayed as static body postures or dynamic movements. Besides, we found slower RTs in participants with ASD, compared to TD ones, specifically in recognizing dynamic stimuli.

### Differences Between Dynamic and Static BM Stimuli

With regard to static body (SB), we did not find any group difference in accuracy and RTs, in line with some previous studies that have investigated the recognition of emotional static body postures in ASD (Doody & Bull, 2013; Libero et al. 2014; Peterson et al. 2015; Weisberg et al. 2014). Similarly, in dynamic stimuli we did not find between groups differences in accuracy, regardless of whether the BM were depicted as solely kinematic information (PLDs) or with visible body form (FLDs), in agreement with previous findings using PLDs in adults with ASD (Actis-Grosso et al. 2015; Murphy et al. 2009; van Boxtel, Dapretto, & Lu, 2016).

The absence of between groups differences in accuracy could be interpreted as the ability to recognise the emotional meaning of observed BM being intact in participants with HFA. Nonetheless, a number of neuroimaging studies have reported comparable behavioural performance, but different brain activation, between participants with and without ASD when presented with BM stimuli (Hubert et al. 2007; Libero et al. 2014; McKay et al. 2012). In line with that, it has been suggested that high-cognitive resources can mediate the acquisition of compensatory strategies that develops with age in individuals with HFA (McKay et al., 2012). According to these studies, an alternative explanation for our failure to find between groups differences in accuracy could be that our participants might have used different—but equally successful—strategies to recognise the emotional meaning of bodily expressions that are mediated by high-cognitive resources and have been developed at a younger age. This alternative explanation would be consistent with research showing that the accuracy in recognizing emotional BM increased with age in children with HFA (Fridenson-Hayo et al. 2016; Mazzoni et al. 2020). However, we acknowledge that in the present study we did not use longitudinal design and thus it was not possible to assess the effective acquisition of compensatory strategies during development. Therefore, this interpretation of our results remains hypothetical and would need to be further investigated. In this regards, longitudinal design would be a very interesting approach for future research as it would help to shed light on the development of emotional BM decoding in individuals with ASD.

Alternatively, another possible explanation for the lack of observed difference in accuracy between the ASD and control groups may be due to the use of the forced-choice response methodology. In TD participants, the forced-choice response has been shown to artificially inflate the rate agreement on emotions expressed by face (Frank & Stennett, 2001) and body posture (Winters, 2005). For instance, Frank and Stennett (2001) showed that, when the correct response was removed, participants tend to respond anyway and to choose an incorrect option at above chance level. The authors suggested that this problem could be resolved by including a "none of the above" label as a response option. In our study, although the correct option was always presented, we did not provide the “none of the above” option and this may have led to artificially inflated the response agreements between the ASD and TD groups. Future studies should hence consider to include the “none of the above” option to avoid this issue.

Concerning RTs, we found an impairment in HFA group specific for the dynamic body stimuli, in line with previous evidence showing that individuals with ASD at all ages struggled in processing the information conveyed by dynamic BM (Atkinson, 2009; Mazzoni et al. 2020; Nackaerts et al. 2012; Philip et al. 2010). In particular, our finding of no group differences in SB, but greater RTs in participants with HFA in recognizing PLDs and FLDs, suggested that individuals with ASD need more time to decode dynamic stimuli compared to TD. Notably, in SB, a single static frame of BM was presented for 3 s, while in dynamic stimuli a sequence of BM was presented within the stimulus duration. Therefore, according to our results, it seems that when the motion information is just implied – as it is in SB – and is presented for a relatively long duration (3 s), the individuals with HFA have enough time to decode the BM emotional meaning and could respond readily as soon as the stimulus disappeared. On the contrary, they seem to need more time to decode dynamic cues from actual motion (FLDs and PLDs videos). This result is consistent with a study on coherent-motion judgment, showing that the performance of adolescents and adults with ASD decreased by shortening the video duration (Robertson et al., 2014). Moreover, our results are in agreement with EMG findings in children with ASD that suggested an impairment in action-chaining mechanism (Cattaneo et al., 2007). This mechanism (Fogassi et al., 2005) has been hypothesised to allow the observer to infer the agent’s intention (Gallese, Fadiga, Fogassi, & Rizzolatti, 2002) and emotions (Jezzini et al., 2015). Consistently, transcranial magnetic stimulation (TMS) studies in humans have provided causal evidence for the specific involvement of parietal areas in processing emotional BM (Engelen et al. 2015, 2018; Mazzoni et al. 2017). Notably, those parietal areas are exactly the brain regions that are believed to underlie the action-chaining mechanism and are an important hub of the putative mirror neurons system (MNS). MNS’ neurons are active both during the execution and the observation of the same action (Rizzolatti et al. 2014) and allow the observer to comprehend an action through the representation of the observed movement within his/her motor system. Interestingly, a number of neuroimaging studies in ASD have showed structural, functional, and connectivity atypicalities in MNS areas during BM perception (Alaerts, Swinnen, & Wenderoth, 2017; Libero et al., 2014; McKay et al., 2012), suggesting that alteration of the MNS areas could explain the difficulty found in BM processing in ASD. In TD individuals, the internal mapping of the observed movement into the observer’s motor system occurs automatically and allows a very rapid recognition of the observed BM (Gallese et al. 2002). For instance, using MEG Meeren et al. (2016) showed a response in right posterior parietal cortex to fearful body postures as early as 80 ms after stimulus onset. Conversely, individuals with HFA may lack this internal simulation and to compensate for it they could develop alternative cognitive strategies that recruit different neural networks (Alaerts et al. 2017; McKay et al., 2012). In fact, although many individuals with HFA can achieve explicit or controlled mentalising skills, the implicit, automatic, and intuitive mechanism for emotion recognition remains impaired even in adulthood (Lai et al. 2014). Thus, although those strategies may allow participants with HFA to recognise the observed BM, they are likely not automatic and would require longer time to achieve BM recognition. In agreement with this, our results showed no group differences in accuracy, but greater RTs in participants with ASD, specifically in recognizing dynamic but not static stimuli.

On these premises, we hypothesise that—similarly to what has been described for grasping (Cattaneo et al. 2007)—our results could be explained by an impaired action-chaining mechanism that would prevent the individuals with ASD to rapidly distinguish the observed BM, e.g. *jump-to-exult* from *jump-to-exercise*, or *rise the arms to self-protect* from *rise the arm to stretch out*. Indeed, in static emotional body posture, only one single movement was displayed. As a consequence, to recognise SB images, the observers did not need to chain any movement because all the information was already available in the observed static posture. Conversely, when the BM stimulus was dynamic, in order to understand its meaning the observer needed to chain together a sequence of movements. This implies that, if the difficulty in ASD was specific to action-chaining, individuals with ASD should present difficulties in recognizing dynamic, but not static stimuli. Notably, this is exactly what we found in the present study.

However, alternative explanations should also be considered. If the use of the forced-choice paradigm did in fact inflate response agreement among the ASD participants, the slower responses in ASD group may reflect their uncertainty about what emotions the stimuli represented. Nonetheless, we found slower responses in ASD participants specifically for dynamic stimuli and this result offers a tentative support for an action-chaining mechanism deficit. Moreover, our results on emotional BM are consistent with previous evidence on static vs dynamic facial expressions. In TD population the motion information seems to facilitate the recognition of facial expressions (Tobin, Favelle, & Palermo, 2016), while previous behavioural and eye tracking studies found atypical responses to facial expressions elicited by dynamic compared to static stimuli in individuals with ASD (Tardif et al. 2007; Uono et al. 2009). Furthermore, reduced facial mimicry in high-functioning ASD was found in responses to dynamic but not to static facial expressions and this reduction was related to social dysfunction (Yoshimura et al. 2015). Finally, fMRI findings showed reduced activation and connectivity of social brain areas in response to dynamic facial expressions in in high-functioning ASD (Pelphrey et al. 2007; Sato et al. 2012). Altogether these studies seem to supports the hypothesis of impaired processing of dynamic emotional faces and are in agreement with our finding on emotional BM.

### Advantage for Fearful Stimuli

Interestingly, in SB we found a significant advantage in recognizing fearful stimuli. Accuracy was higher in fearful SB than neutral and (marginally) happy SB in both groups. The fearful-advantage has been previously described in behavioural studies on recognition of emotional BM both in individuals with TD (Atkinson et al. 2004; Bannerman et al. 2009) and ASD (Mazzoni et al., 2020; Philip et al., 2010). From an evolutionary point of view, the vision of an emotional expression triggers adaptive actions (Darwin, 1872). Frijda wrote that "Emotions exist for the sake of signalling states of the world that have to be responded to or that no longer need response and action” (Frijda, 1988, p.354). In other words, this author suggests that the emotions exist for the sake of action, for dealing with the environment, and highlighted that different emotions arise in response to different situation and prompt different reaction (Frijda, 1988; Frijda et al. 1989). Some emotions are more relevant when the observer is close to the agent (e.g., disgust or happiness) (Gelder et al. 2015), as they are aimed to trigger a behavioural response over a close source of emotion (e.g. throwing away a bad food, or getting close to something pleasant in order to enjoy it). Those emotions are more likely to be expressed and recognised better through the face, which indeed requires proximity to be perceived. Instead, some other emotions are expressed and recognised better with BM (e.g. threatening signals, such as fear and anger, e.g. Actis-Grosso et al., 2015). Those emotions communicate the presence of threats or dangers in the environment, hence their recognition is as more important for survival as they can be seen from a distance. Indeed, this enables the observer to have “enough” time for reacting promptly and adaptively (e.g. fight or flight), maximizing the chance of survival. In particular, fearful BM perception is associated with increased vigilance and attention (Bannerman et al. 2009; Kret et al. 2013; Phelps et al. 2006; Tamietto et al. 2007), improved visual processing (Borhani et al. 2015; van Heijnsbergen, Meeren, Grèzes, & de Gelder, 2007), and enhanced reactivity of the motor system (Borgomaneri, Gazzola, & Avenanti, 2012; Borgomaneri et al. 2015). Due to its critical evolutionary salience, in our opinion, it is not completely surprising that we found a fear-advantage also in individuals with high-functioning ASD.

Our results are partially in contrast with some findings that posited a deficit in decoding fearful signals in ASD, possibly related to dysfunctions in amygdala (Ashwin, Chapman, Colle, & Baron-Cohen, 2006; Hadjikhani et al., 2009; Howard et al. 2000; Schultz, 2005). However, it is important to acknowledge that our sample includes people with HFA, who might have developed compensatory mechanisms to recognise the social relevant stimuli—especially when evolutionary vital—at a TD-level.

Moreover, our stimuli depicted the emotional expressions at their peak intensity, possibly being too easily recognizable for participants with HFA. Finally, in the present study participants were asked to perform a low demanding experimental task. Indeed, it has been showed that, when the task is complex, the performance of participants with ASD might be hampered by their difficulties with attention and working memory (Barendse et al., 2013; Happé, Ronald, & Plomin, 2006). Therefore, to prevent the results from reflecting the task demand instead of the emotion recognition ability, we minimised the cognitive demands by presenting only three emotional contents. This allowed us to limit the number of response options (i.e. working memory) and the duration of the experiment (i.e. attention). Since we tested individuals with HFA, there is the possibility that we obtained a ceiling effect in the accuracy because the task was actually too simple for our participants.

To unmask differences in accuracy in recognizing the emotional expression in individuals with HFA that have been in treatment for years, future studies aimed at i) presenting the stimuli briefly, ii) using subtler expressions, and iii) using more complex tasks—e.g. with increased number of presented emotions and response options- are desirable.

As a final comment, we acknowledge the small sample size as a major limit of the study that necessarily impose caution in interpreting our results. Historically, the field of emotional BM recognition in ASD has been characterised by small-scale studies that have yielded to contradictory results. In fact, small sample size may have only partially represented the ASD population as it is characterised by a well-recognized heterogeneity in functioning and clinical profile. Although in our statistical analyses we tried to account for the individual variability, it is important to highlight that our limited sample size does not allow to draw robust conclusions. This limitation is remarkable and future research is needed to corroborate our findings. Nevertheless, the present study added some novelty to the field as it explored a new aspect of bodily emotion recognition in ASD and showed an interesting – although preliminary—differences between static and dynamic body stimuli. Our results could hence serve as an interesting starting point for future research that should necessarily involve larger sample size in order to account for heterogeneity in ASD.

## Conclusions and Implication for Treatment

To conclude, our results showed similar accuracy but slower RTs between TD and HFA participants in recognizing dynamic but not static BM. The TD-like accuracy suggests that individuals with HFA may develop alternative—but equally successful—high cognitive strategies to recognise the emotional meaning of BM that are likely mediated by different neural networks. However, those strategies are not automatic and require longer time to achieve BM recognition. Noteworthy, despite no differences in accuracy, our results showed slower RTs in processing dynamic BM in individuals with HFA. We interpreted this result in light of atypicalities in MNS that may yield to deficit in action chaining mechanism and prevent the individuals with ASD to infer automatically and rapidly the emotional content of dynamic body movements. Finally, we found a significant advantage in recognizing fearful SB in both TD and HFA groups. This could be interpreted either as the processing of evolutionary salient stimuli being intact in individuals with HFA or, alternatively, as our participants having compensated their difficulties in understanding emotional bodily information thanks to the received intervention. These findings have important implications for treatment. Indeed, people with ASD might find it challenging to make eye contact or to look at faces at close distance (Tanaka & Sung, 2016). Contrarily, the recognition of BM could be less troublesome because it can be viewed at “safer” interpersonal distance than faces. Therefore, along with emotional facial and vocal training, the interventions in individuals with ASD should also focus on improving automaticity of BM recognition. This should be a component of a broader-based social skills treatment program that, in turn, might help individuals with ASD to better infer feelings and intentions of other people and to improve social interactions.

## Supplementary Information

Below is the link to the electronic supplementary material.

Supplementary file1 (DOCX 23 KB)
