# Supplementary material for: Difficulties in Recognising Dynamic but not Static Emotional Body Movements in Autism Spectrum Disorder
Source: J Autism Dev Disord. 2021 Apr 17;52(3):1092–105. doi: 10.1007/s10803-021-05015-7 (PMC8854267; doi:10.1007/s10803-021-05015-7)
Supplement: Supplementary file 1 — Supplementary file1 (DOCX 23 KB) [file 10803_2021_5015_MOESM1_ESM.docx]

**Supplementary materials**

**Creation of Static body images (SB) dataset**

The set of static images were created by our group of research. Eight actors (4 females and 4 males) were asked to portray four types of emotions (Happiness, Fear, Sadness, and Anger) and a number of neutral actions whose motion quality and intensity were similar to that of emotional expressions (i.e., kick, pick up something from the floor, wear a sock, run, push). For the emotional expressions, the actors received examples of potential scenarios, such as joyful (e.g. your team has just scored and won the final match) or threatening (e.g. a giant lion is running over you) situations. In order to reduce any possible biases related to the actors’ clothes, they wore a white T-shirt with long sleeves, blue jeans, and sneakers. All the pictures were edited with Photoshop: brightness, number of pixels and dimension were equalised among all the pictures, the background color was turned to black, and the face was blurred. We initially selected 12 pictures for each of the emotional expressions and neutral actions, for a total 48 emotional and 60 neutral images.

**Validation study:** This set of images was presented to 20 TD adults (16 males and 4 females, age M = 24.65, age SD = 1.66) to assess the effective recognizability of the stimuli. The male:female ratio was chosen to equalise the 4:1 prevalence reported in ASD (DSM-5 p.57; Fombonne, 2009). The software E-Prime 2.0 was used to present the stimuli and to collect the responses (accuracy and response times). Every trial started with one-second fixation cross, then the image appeared centrally and lasted for a maximum of 5 seconds. The emotional and neutral images were presented in two separated blocks (Emo and Neutral). In the block “Emo”, participants were asked i) to recognise the emotional content of the observed BM and ii) to rate its intensity. In the block “Neutral” participants were asked i) to recognise the type of neutral actions and ii) to judge whether the presented action was or not an emotion. In the block Emo, the question “Which emotion is expressed by the actor?” appeared together with the image on the top of the screen, and 5 response options were presented on the bottom part of the screen (Fear, Happiness, Anger, Sadness, plus the option Other). Responses were collected by keyboard. A sticky label with the initial of every response option was placed on the response keys (i.e. the keys “F”, “G”, “H”, “J”, “K”). The correspondence between keys and response options were randomised across participants. Participants responded using their right hand. After the participants’ response, the question “How intense was the expressed emotion?” was displayed together with a 7-points rating scale (where 1 corresponded to “not intense at all” and 7 to “extremely intense”). To rate the images, participants were asked to use the keys with numbers from 1 to 7. A similar procedure was used also in the block Neutral. The image was presented centrally together with the question “Which actions is performed by the actor?” that appeared on the top of the screen, and the 6 response options that were presented on the bottom part of the screen (the five abovementioned actions, plus the option “Other”). Following the participants’ response, a second slides with the question “Was the presented action an emotional expression?” appeared and participants were asked to answer “Yes” or “No” by pressing the corresponding keys (“B” or “N”) on the keyboard. The order of the two blocks was counterbalanced between participants. Within each block the images were presented randomly.

In the main study, we included only stimuli depicting happy, fearful, and neutral movements. We selected the stimuli whose accordance about the expressed emotion ranged between 80 and 100% (that is, at least 80% of participants responded that that images depicted a specific emotional expression - e.g., fear - or a specific action - e.g., push). For the emotional expressions, we selected only the fearful and happy stimuli whose intensity was rated above 4.7; for the neutral actions, we selected only the stimuli that were never confounded as an emotion. According to these criteria, a total of 24 images were selected (12 happy, 12 fearful, and 12 neutral). The actors’ gender was equally represented (4 males and 4 females). The final dataset of SB images is available on request from the corresponding author.

**Percentage of Accuracy, subdivided by groups (first row), class of stimuli (columns), and emotional category (rows).**

|  | **Total** | | | **ASD** | | | **TD** | | |
| --- | --- | --- | --- | --- | --- | --- | --- | --- | --- |
|  | *Static Body (BS)* | *FLDs* | *PLDs* | *Static Body (BS)* | *FLDs* | *PLDs* | *Static Body (BS)* | *FLDs* | *PLDs* |
| *Fear* | 0.97 | 0.96 | 0.94 | 0.98 | 0.97 | 0.93 | 0.96 | 0.95 | 0.94 |
| *Happiness* | 0.94 | 0.92 | 0.90 | 0.95 | 0.92 | 0.92 | 0.94 | 0.92 | 0.87 |
| *Neutral* | 0.94 | 0.97 | 0.96 | 0.94 | 0.97 | 0.95 | 0.95 | 0.96 | 0.97 |

**Results of the analysis of Deviance Table (Anova with Type III Wald chi-square tests) of the generalised linear mixed model computed separately in the three types of stimuli (top row):**

Accuracy

|  | ***BS*** | | | ***FLDs*** | | | ***PLDs*** | | |
| --- | --- | --- | --- | --- | --- | --- | --- | --- | --- |
|  | χ^2^ | *Df* | *p-value* | χ^2^ | *Df* | *p-value* | χ^2^ | *Df* | *p-value* |
| ***Emotion*** | *8.405* | *2* | *0.015** | *2.99* | *2* | *0.224* | *3.62* | *2* | *0.164* |
| ***Group*** | *0.035* | *1* | *0.851* | *1.33* | *1* | *0.248* | *0.038* | *1* | *0.846* |
| ***Emotion*Group*** | *0.506* | *2* | *0.776* | *1.845* | *2* | *0.397* | *3.238* | *2* | *0.198* |

Reactions Times:

|  | ***BS*** | | | ***FLDs*** | | | ***PLDs*** | | |
| --- | --- | --- | --- | --- | --- | --- | --- | --- | --- |
|  | χ^2^ | *Df* | *p-value* | χ^2^ | *Df* | *p-value* | χ^2^ | *Df* | *p-value* |
| ***Emotion*** | 0.567 | *2* | *0.753* | *2.263* | *2* | *0.322* | *2.713* | *2* | *0.257* |
| ***Group*** | *0.949* | *1* | *0.329* | *3.833* | *1* | *0.050** | *9.063* | *1* | *0.003*** |
| ***Emotion*Group*** | *0.309* | *2* | *0.859* | *0.001* | *2* | *0.999* | *2.728* | *2* | *0.255* |

**Comparisons between the linear mixed models of reaction times (LogRT) in FLDs without and with IQ as covariate (first and second row, respectively).**

|  | **Df** | **AIC** | **BIC** | **logLik** | **deviance** | χ^2^ | **Df** | **p-value** |
| --- | --- | --- | --- | --- | --- | --- | --- | --- |
| **Model without IQ** | 13 | 2861.00 | 2934.68 | -1417.500 | 2835.00 |  |  |  |
| **Model with IQ** | 14 | 2862.56 | 2941.90 | -1417.279 | 2834.56 | 0.442 | 1 | 0.5059704 |

The result shows that the two models are not significantly different**.**
